# Increased Coffee Intake Reduces Circulating HBV DNA and HBsAg Levels in HBeAg-Negative Infection: A Cohort Study

**DOI:** 10.3390/v11090808

**Published:** 2019-09-01

**Authors:** Jack Bee Chook, Yun Fong Ngeow, Kok Keng Tee, Jamie Wan Ting Lee, Rosmawati Mohamed

**Affiliations:** 1 Department of Medical Sciences (DMS), School of Healthcare and Medical Sciences (SHMS), Sunway University, Bandar Sunway, Selangor Darul Ehsan 47500, Malaysia; 2 Department of Pre-Clinical Sciences, Faculty of Medicine and Health Sciences, Universiti Tunku Abdul Rahman, Bandar Sungai Long, Selangor 43000, Malaysia; 3 Department of Medical Microbiology, Faculty of Medicine, University of Malaya, Kuala Lumpur 50603, Malaysia; 4 Department of Medicine, Faculty of Medicine, University of Malaya, Kuala Lumpur 50603, Malaysia

**Keywords:** hepatitis B virus, coffee, viral load, surface antigen, antiviral

## Abstract

Coffee is hepatoprotective and potentially antiviral; however, its anti-hepatitis B virus (anti-HBV) property is not known in humans. This study investigated the influence of coffee drinking behaviour as well as clinical and biochemical profiles of hepatitis B e antigen (HBeAg) negative participants on circulating HBV DNA and hepatitis B surface antigen (HBsAg) levels at a 24-week interval. Exactly 114 chronically HBV-infected adult participants were enrolled from the University of Malaya Medical Centre (UMMC), Malaysia. A significant reduction of HBV DNA level was observed in those drinking three or more cups of coffee per day, with a median reduction of 523 IU/mL (*P* = 0.003). Reduction of HBsAg level was observed in those drinking two cups per day, with a median reduction of 37 IU/mL (*P* < 0.001). Multivariate analysis showed that increased coffee intake (*P* = 0.015) and lower ALT level (*P* = 0.033) were the significant predictors for a lower HBV DNA level, whereas increased coffee intake (*P* = 0.002) and having a family history of HBV infection (*P* = 0.021) were the significant predictors for a lower HBsAg level. These data suggest that drinking three cups or more coffee per day reduces circulating HBV DNA and HBsAg levels.

## 1. Introduction

Coffee, one of the most common beverages consumed worldwide, is known to be the source of bioactive compounds beneficial to human health. It reduces the risk of malignancies such as liver, renal and breast cancers [1,2,3], is associated with a lower risk of elevated liver enzyme activity [4], less liver fibrosis and inflammation in non-alcoholic fatty liver disease, hepatitis B (HBV) and hepatitis C (HCV) [5], and lower mortality from chronic liver disease in male smokers [6]. The anti-HBV effects of coffee, however, appears not to have been investigated in any human studies.

Coffee is a complex mixture of proteins, carbohydrates, lipids, potassium, magnesium, and antioxidants such as caffeine, chlorogenic acids and polyphenols [7]. Chlorogenic acid and its metabolised products (caffeic and quinic acids) are likely responsible for the cancer-protective effects and anti-inflammatory properties of coffee [8,9,10]. 

Currently, the mainstay therapy for hepatitis B consists of immunomodulators and nucleos(t)ide analogues. These medications suppress viral replication and disease progression but are not a functional cure for chronic hepatitis B. Keeping circulating HBV DNA and HBsAg levels low in the long term may reduce the risk of progression to severe liver diseases [11]. In this regard, we conducted a prospective cohort study to investigate possible anti-HBV effects of coffee in humans. We examined host coffee drinking behaviour, biochemical and blood cell profiles, to determine their influence on circulating HBV DNA and HBsAg levels in chronically HBV-infected individuals. 

## 2. Materials and Methods

### 2.1. Study Participants

We prospectively enrolled 114 chronically HBV-infected adult participants from the University of Malaya Medical Centre (UMMC) in Kuala Lumpur, Malaysia. Written informed consent was obtained from each participant. This study was approved by the UMMC Medical Ethics Committee (reference number: 961.1), acting by the ethical standards of the Declaration of Helsinki. Chronic HBV infection was diagnosed in patients who were HBsAg positive for at least 6 months. All participants tested normal for serum alanine aminotransferase (ALT) and were test-negative for HBeAg. The exclusion criteria included severe liver diseases like liver cirrhosis and hepatocellular carcinoma, as evidenced by ultrasonography, transient elastography (FibroScan, Paris, France) and radiography, alcoholics, and heavy tea drinkers, in whom, the presence of polyphenols from tea might confound the interpretation of study results.

### 2.2. Assessment of Exposure and Clinical Data

A bilingual (English and Malay) questionnaire was used to gather information on demography, coffee drinking habit, cigarette smoking, alcohol drinking, history of antiviral treatment, and family history of chronic HBV infection. Five of the participants were chronic smokers having >15 cigarettes/day. All participants were treatment naïve, except for two who had past antiviral treatment but have not been treated for more than one year before entering this study. At the baseline (first visit), participants were asked to do their own recording of daily coffee consumption and feed back to us on the next follow up (second visit). This was to minimise the risk of recall bias. Their liver function and blood cell profiles were assessed at two time-points (baseline and second visit, separated by a median interval of 24 weeks) 

### 2.3. Virological Tests

Viral activity was measured by HBV DNA and HBsAg levels. Plasma HBV DNA was determined by the Roche Cobas Taqman assay (Roche Molecular System Inc., (Branchburg, NJ, USA). Serum HBsAg level was measured by the Elecsys(R) HBsAg II Quant assay (Roche Diagnostics, Mannheim, Germany). Both HBV DNA and HBsAg levels were measured at baseline and follow-up visits. 

### 2.4. Calculation of Ratio

For each variable, a ratio was calculated by dividing the value at the second visit over the value at baseline (the first visit), to indicate fold change. For HBV DNA (designated as Group A), a ratio of less than 1 was assigned as Group A1, whereas that of more than 1 was assigned as Group A2. Similarly, for HBsAg (designated as Group B), a ratio of less than 1 was assigned as Group B1, whereas that of more than 1 was assigned as Group B2. A higher ratio implies an increase in level during the interval between the two time-points and vice versa. To investigate the antiviral effects of coffee, the participants were categorised into four groups based on their coffee consumption: Group 0, coffee abstainer; Group 1: one cup per day; Group 2: two cups per day; and Group 3: three or more cups per day. A cup of coffee was defined as at least one teaspoon of coffee powder.

### 2.5. Statistical Analysis

Statistical analysis was performed using SPSS 25 (SPSS Inc., Chicago, USA). The Chi-squared exact test was used for categorical variables, whereas the Spearman rho correlation, Mann–Whitney U and the Wilcoxon Signed Ranks tests were used for continuous variables, where appropriate. Odds ratio and 95% confidence interval (CI) were calculated for categorical variables. Median and interquartile range (IQR) for continuous variables, where appropriate. Multivariate-adjusted odds ratios were calculated using logistic regression model. Two-sided P values of less than 0.05 were taken as significant.

## 3. Results

### 3.1. Factors Influencing HBV DNA

After a median follow-up of 24 weeks, two-point data were successfully collected from 114 chronically HBV-infected participants, of whom, 60.5% (69/114) were in Group A1 (HBV DNA ratio <1) and 39.5% (45/114) in HBV DNA Group A2. These two groups were comparable in age and gender. With univariate analysis, the HBV DNA ratio was lower in those with higher coffee intake (*P* = 0.003), as well as lower ratios of ALT (*P* = 0.027) and lymphocyte count (*P* = 0.014) (Table 1). Age- and gender-adjusted multivariate analysis showed that participants with higher coffee intake and lower ALT ratio were significant predictors for lower HBV DNA ratio.

### 3.2. Factors Influencing HBsAg

In HBsAg group, 64.9% (74/114) were in group B1 and 35.1% (40/114) in group B2. These two groups were comparable in age and gender. Univariate analysis showed that the HBsAg ratio to be higher in participants with lower coffee intake (*P* = 0.003) and without a family history of HBV infection (*P* = 0.010) (Table 2). Age- and gender-adjusted multivariate analysis showed that participants with higher coffee intake and with a family history of HBV infection were significant predictors for lower HBsAg ratio (Table 3).

### 3.3. Influence of Coffee Drinking Habit on HBV DNA, HBsAg and ALT

In the present study, 31.6% (36/114) participants were coffee abstainers, 14.9% (17/114) were drinking one cup of coffee per day, 36.8% (42/114) two cups per day and 16.7% (19/114) at least three cups per day (Table 4). Using the Wilcoxon Signed Ranks test, a significant reduction in HBV DNA level was observed only in participants drinking three or more cups of coffee per day (*P* = 0.003). The median reduction of HBV DNA in those drinking at least three cups of coffee per day was 523 IU/mL. Significant HBsAg reduction was seen in those drinking two cups of coffee per day (*P* < 0.001) for two cups per day and for three or more cups per day (*P* = 0.033). The median reduction of HBsAg in those drinking two or at least three cups of coffee per day was 37 IU/mL and 11 IU/mL, respectively. No significant difference in ALT was observed in participants drinking two and at least three cups of coffee per day (*P* = 0.989 and 0.187, respectively). There was moderate inverse correlation between drinking coffee and baseline/follow-up HBsAg (Spearman rho, *r* = −0.262, *P* = 0.005; *r* = −0.279, *P* = 0.003), but this was not observed between drinking coffee and baseline/follow-up HBV DNA (*r* = 0.032, *P* = 0.733; *r* = −0.047, *P* = 0.619).

### 3.4. Family History of HBV Infection, HBV DNA and HBsAg

Out of 114 participants, 54.4% (62/114) participants had a family history of chronic HBV infection and 45.6% (52/114) did not have. Using the Wilcoxon Signed Ranks test, a significant reduction in the HBsAg level was observed in participants with a family history of chronic HBV infection (*P* < 0.001) but not in those without the family history (*P* = 0.735). There was no significant reduction in the HBV DNA level in those with (*P* = 0.089) and without (*P* = 0.885) a family history of chronic HBV infection.

## 4. Discussion

Our study results showed an association between increased coffee intake and reduction in both circulating HBV DNA and HBsAg levels in humans with chronic hepatitis B, at a median interval of a 24-week period. Improvement in ALT level predicts reduction in the HBV DNA level, whereas a family history of chronic HBV infection is associated with a decrease in the HBsAg level.

Many studies, inclusive of case-control and cohort studies, have demonstrated an inverse relationship between coffee consumption and liver cirrhosis [12,13] and hepatocellular carcinoma (HCC) [14,15,16]. Individuals having greater HBV DNA levels are at an increased risk of developing liver cirrhosis and HCC [17,18], and that long-term suppression of HBV DNA decreases hepatic fibrosis, reverses cirrhosis and reduces the development of HCC [19]. These observations suggest that coffee may potentially reduce the HBV DNA level. Polyphenols (like chlorogenic and caffeic acids), but not caffeine, in coffee have been shown, in cell culture and animal studies, to inhibit the replication of HBV [20]. Indeed, this anti-HBV activity of coffee is implied in our results as chronic hepatitis B participants taking at least three cups of coffee per day had a significant reduction in their circulating HBV DNA levels compared to those drinking less, and those taking at least two cups of coffee per day had a significant reduction in their circulating HBsAg levels. These effects remained significant even after multivariate-adjusted analysis. Coffee drinking did not produce any obvious improvement in ALT levels, probably because all the participants had normal ALT levels at the time of entry into this study. 

A family history of chronic HBV infection is a unique risk factor for naturally-occurring early-onset HCC patients with HBV-associated liver cirrhosis [21]. It has also been associated with the resectability of liver tumours [22]. Hence, a family history of the infection seems to have some impact on the course and clinical management of chronic hepatitis B. In general, HBsAg decreases as a chronic hepatitis B person gets older [23]. It seems that the greater the length of time a person is infected by HBV, the better is their immunological control over the viral antigen, though the mechanism remains unknown. However, the immunological control does not appear to apply to the viral multiplication in liver cells. This is reflected in our results in participants with a family history of chronic HBV infection that show lower HBsAg levels, but not HBV DNA levels.

This study has several recognised strengths and limitations. Our prospective approach reduces recall inaccuracy, as the participants reported their current status of coffee intake. The limitations were this was a single centre study and the sample size was small. We did not take into account various types of dietary consumption including polyphenol-rich food like fruits, red wine, beer and cocoa. In addition, our observations could be confounded by other factors. For example, milk consumption may reduce the intestinal absorption of chlorogenic acid in coffee [24]. In this case, a person with chronic hepatitis B who drinks coffee to which milk is added may need to drink more cups of coffee in order to achieve the same antiviral effect. The choice of coffee types and different ways of preparation could also contribute to data variation to a certain extent, as different types of coffee may have different compositions of polyphenols [25,26]. 

In conclusion, our findings suggest that HBV-infected individuals drinking coffee at a rate of three cups or more per day are more likely to experience reduction in both circulating HBV DNA and HBsAg levels. This proposition supports the observations of several large sample size cohorts that coffee consumption at three cups or more per day is needed to reduce the risk of HCC [14,27]. Drinking coffee may provide chronically HBV-infected individuals an alternative way to keep the virus inactive and maintain their liver health in the long term. This hypothesis, if further strengthened by randomised controlled studies using a standard type and preparation of coffee or its ingredients, could prove beneficial to chronically HBV-infected individuals.

## Figures and Tables

**Table 1:** Effects of demography, liver function, blood cell profile and HBsAg level on HBV DNA level by univariate analysis.

Characteristics	Group A1 (*N* = 69)	Group A2 (*N* = 45)	OR (95% CI)	*P* Value
Age	57.0 (46.5–65.5)	53.0 (44.5–63.5)		0.417
Male	28	21	1.281 (0.601–2.733)	0.565
Smoker	3	2	1.023 (0.164–6.379)	1.000
Family history of HBV infection	39	23	0.804 (0.378–1.709)	0.701
Coffee intake (cups/day)	2 (0–3)	1 (0–2)		0.007
Liver function ratio				
Albumin	1.000 (0.977–1.045)	1.000 (0.978–1.045)		0.941
Total bilirubin	1.000 (0.840–1.268)	1.000 (0.800–1.143)		0.156
Conjugated bilirubin	1.000 (0.817–1.250)	1.000 (0.750–1.000)		0.103
ALT	0.938 (0.783–1.170)	1.022 (0.882–1.175)		0.027
AST	0.958 (0.884–1.051)	1.000 (0.897–1.123)		0.116
GGT	1.000 (0.898–1.137)	1.000 (0.889–1.142)		0.997
Alkaline phosphatase	0.982 (0.887–1.038)	0.987 (0.902–1.069)		0.691
Blood cell count ratio				
Platelet	1.007 (0.922–1.083)	1.054 (0.976–1.116)		0.070
Neutrophil	0.964 (0.797–1.059)	1.017 (0.841–1.106)		0.380
Lymphocyte	0.964 (0.883–1.110)	1.064 (0.943–1.205)		0.014
Monocyte	0.939 (0.788–1.088)	1.010 (0.852–1.112)		0.117
Eosinophil	1.050 (0.846–1.423)	1.077 (0.883–1.238)		0.848
Virological profile				
HBsAg level ratio	0.937 (0.780–1.070)	0.926 (0.814–1.041)		0.835

Abbreviations: ALT, alanine aminotransferase; AST, aspartate aminotransferase; GGT, gamma glutamyl transferase; OR, odds ratio. All continuous data are expressed in median and interquartile range (IQR). Group A1: HBV DNA ratio <1; Group A2: HBV DNA ratio >1. HBV DNA ratio was calculated based the value at second visit over the value at first visit for a median interval period of 24 weeks.

**Table 2:** Effects of demography, liver function, blood cell and virological profiles on HBsAg level by univariate analysis.

Characteristics	Group B1 *N* = 74	Group B2 *N* = 40	OR (95% CI)	*P* Value
Age	55.0 (45.0–63.0)	60.0 (49.8–66.0)		0.098
Male	32	17	0.970 (0.446–2.111)	1.000
Smoker	3	2	1.246 (0.199–7.781)	1.000
Family history of HBV infection	47	15	0.345 (0.155–0.764)	0.010
Coffee intake (cups/day)	2 (0–2)	1 (0–2)		0.003
Liver function ratio				
Albumin	1.000 (0.977–1.045)	1.000 (0.979–1.044)		0.741
Total bilirubin	1.000 (0.833–1.250)	1.000 (0.794–1.204)		0.948
Conjugated bilirubin	1.000 (0.800–1.200)	1.000 (0.788–1.031)		0.810
ALT	1.000 (0.824–1.198)	0.934 (0.806–1.110)		0.553
AST	1.000 (0.876–1.129)	0.926 (0.868–1.011)		0.152
GGT	1.000 (0.910–1.157)	1.000 (0.850–1.100)		0.564
Alkaline phosphatase	0.976 (0.889–1.045)	0.995 (0.891–1.064)		0.605
Blood cell count ratio				
Platelet	1.019 (0.950–1.084)	1.038 (0.977–1.104)		0.170
Neutrophil	0.970 (0.806–1.048)	1.013 (0.860–1.367)		0.202
Lymphocyte	1.020 (0.908–1.166)	0.985 (0.888–1.158)		0.776
Monocyte	0.941 (0.801–1.068)	1.025 (0.851–1.144)		0.148
Eosinophil	1.061 (0.857–1.405)	1.067 (0.803–1.242)		0.917
Virological profile				
HBV DNA level ratio	1.000 (0.455–1.856)	0.576 (0.301–1.490)		0.444

Abbreviations: ALT, alanine aminotransferase; AST, aspartate aminotransferase; GGT, gamma glutamyl transferase; HBV, hepatitis B virus; HBsAg, hepatitis B surface antigen; OR, odds ratio. All continuous data are expressed in median and interquartile range (IQR). Group B1: HBsAg ratio < 1; Group B2: HBsAg ratio > 1. HBsAg ratio was calculated based the value at second visit over the value at first visit for a median interval period of 24 weeks.

**Table 3:** Logistic regression modeling based on HBV DNA and HBsAg grouping.

Characteristics	Multivariate Analysis		
	S.E.	Adjusted OR (95% CI)	*P* Value
Group A: HBV DNA			
Age	0.017	0.991 (0.958–1.025)	0.583
Gender (female as reference)	0.417	1.233 (0.544–2.793)	0.616
Coffee intake (cups/day)	0.198	0.618 (0.420–0.911)	0.015
ALT ratio	0.627	3.805 (1.114–12.995)	0.033
Lymphocyte count ratio	1.022	6.041 (0.815–44.791)	0.079
Group B: HBsAg			
Age	0.019	1.035 (0.997–1.074)	0.069
Gender (female as reference)	0.464	0.703 (0.283–1.746)	0.703
Coffee intake (cups/day)	0.209	0.530 (0.352–0.798)	0.002
Family history of HBV infection	0.459	0.348 (0.141–0.856)	0.021

Abbreviations: ALT, alanine aminotransferase; HBV, hepatitis B virus; OR, odds ratio; S.E., standard error. Group A - A1: HBV DNA ratio <1; A2: HBV DNA ratio >1; A1 as reference group. Group B - B1: HBsAg ratio <1; B2: HBsAg ratio >1; B1 as reference group. The ratios were calculated based the value at second visit over the value at first visit for a median interval period of 24 weeks.

**Table 4:** Demography, clinical and virological characteristics of coffee drinkers.

Characteristics	Coffee Intake (Cups per Day)			
	0 (*N* = 36)	1 (*N* = 17)	2 (*N* = 42)	≥3 (*N* = 19)
Age	56 (47–62)	55 (43–62)	56 (47–69)	60 (49–66)
Male	15	9	19	6
Smoker	4	0	1	0
Family history of HBV infection	16	11	24	11
**Liver function**				
Albumin, g/L Baseline, Follow-up	44 (43–45) 45 (44–47)	45 (43–46) 45 (42–46)	44 (41–45) 44 (43–45)	45 (43–47) 45 (44–46)
Total bilirubin, µmol/L Baseline, Follow-up	11.5 (9.0–15.0) 11.0 (9.0–15.0)	13.0 (11.0–20.0) 13.0 (10.0–19.0)	12.0 (10.0–15.8) 11.0 (9.0–15.0)	10.0 (7.5–12.0) 11.0 (9.5–14.5)
Conjugated bilirubin, µmol/L Baseline, Follow-up	3.5 (3.0–5.0) 4.0 (3.0–4.3)	5.0 (3.0–6.0) 5.0 (3.0–5.0)	4.0 (3.0–4.0) 3.0 (3.0–4.8)	3.0 (3.0–4.0) 3.0 (3.0–4.5)
ALT, U/L Baseline, Follow-up	24.0 (19.0–31.3) 22.0 (16.8–28.3)	23.0 (19.0–29.0) 21.0 (16.0–22.0)	23.5 (17.0–31.8) 22.0 (17.3–29.5)	20.0 (16.5–29.0) 18.0 (16.0–26.0)
AST, U/L Baseline, Follow-up	23.0 (20.0–27.5) 22.0 (19.8–25.0)	22.0 (19.0–26.0) 21.0 (18.0–24.0)	24.0 (20.0–26.0) 23.0 (20.0–26.8)	23.0 (21.5–28.0) 23.0 (21.0–26.0)
GGT, U/L Baseline, Follow-up	20.0 (14.0–24.0) 17.5 (13.8–26.3)	20.0 (15.0–38.0) 21.0 (16.0–41.0)	20.5 (17.0–27.8) 20.0 (15.3–29.8)	18.0 (13.5–24.5) 17.0 (12.5–24.0)
Alkaline phosphatase, U/L Baseline, Follow-up	75.0 (63.3–94.5) 74.0 (62.0–90.5)	67.0 (58.0–80.0) 69.0 (63.0–79.0)	76.5 (66.0–92.5) 74.0 (67.0–93.8)	74.0 (58.5–80.0) 65.0 (56.5–77.0)
**Blood cell count**				
Platelet, × 10^9^/L Baseline, Follow-up	240 (217–267) 245 (218–292)	258 (219–270) 280 (208–296)	254 (214–288) 256 (217–313)	259 (220–284) 256 (219–271)
Neutrophil, × 10^9^/L Baseline, Follow-up	3.27 (2.91–4.02) 3.23 (2.37–4.26)	2.97 (2.22–3.21) 3.19 (2.73–4.75)	4.03 (3.02–4.79) 3.91 (2.89–4.29)	3.27 (2.74–4.03) 2.85 (2.47–3.59)
Lymphocyte, × 10^9^/L Baseline, Follow-up	1.83 (1.55–2.23) 2.00 (1.68–2.37)	1.61 (1.45–1.79) 1.56 (1.32–2.15)	2.18 (1.90–2.63) 2.24 (1.81–2.56)	1.62 (1.35–2.01) 1.50 (1.33–2.03)
Monocyte, × 10^9^/L Baseline, Follow-up	0.44 (0.36–0.57) 0.44 (0.35–0.51)	0.39 (0.30–0.53) 0.43 (0.30–0.61)	0.49 (0.39–0.69) 0.45 (0.37–0.56)	0.44 (0.37–0.54) 0.40 (0.38–0.45)
Eosinophil, × 10^9^/L Baseline, Follow-up	0.16 (0.07–0.26) 0.16 (0.11–0.31)	0.15 (0.08–0.24) 0.18 (0.06–0.21)	0.15 (0.09–0.22) 0.13 (0.09–0.23)	0.15 (0.10–0.27) 0.18 (0.11–0.28)
**Virological profile**				
HBsAg, IU/mL Baseline, Follow-up	581.70 (158.20–1,294.25) 622.50 (146.25–1,630.25)	378.80 (20.44–1,642.00) 375.00 (23.00–1,252.00)	175.50 (24.84–605.15) 138.00 (15.00–600.25)	93.75 (3.20–365.80) 83.00 (2.00–320.00)
HBV DNA, IU/mL Baseline, Follow-up	781.5 (141.3–4,153.3) 624.0 (118.5–2,723.8)	540.0 (135.0–2,567.0) 925.0 (119.0–3,109.0)	622.5 (71.3–4,120.8) 353.0 (88.3–4,121.8)	937.0 (177.0–5,445.0) 414.0 (34.5–2,846.5)

ALT, alanine aminotransferase; AST, aspartate aminotransferase; GGT, gamma-glutamyl transferase; HBsAg, hepatitis B surface antigen. All continuous variables are expressed in median and interquartile range (IQR). The P values for comparison of various characteristics between baseline and follow-up are as follows: Age, *P* = 0.434; Male, *P* = 0.630; Smoker, *P* = 0.228; Family history of HBV infection, *P* = 0.507; Albumin, *P* = 0.114 (0), *P* = 0.895 (1), *P* = 0.122 (2), *P* = 0.528 (≥3); Total bilirubin, *P* = 0.596 (0), *P* = 0.419 (1), *P* = 0.361 (2), *P* = 0.107 (≥3); Conjugated bilirubin, *P* = 0.569 (0); *P* = 0.427 (1), *P* = 0.680 (2), *P* = 0.126 (≥3); ALT, *P* = 0.176 (0); *P* = 0.046 (1), *P* = 0.989 (2), *P* = 0.187 (≥3); AST, *P* = 0.718 (0); *P* = 0.072 (1), *P* = 0.883 (2), *P* = 0.659 (≥3); GGT, *P* = 0.873 (0), *P* = 0.585 (1), *P* = 0.732 (2), *P* = 0.310 (≥3); Alkaline phosphatase, *P* = 0.066 (0); *P* = 0.407 (1), *P* = 0.343 (2), *P* = 0.014 (≥3); Platelet, *P* = 0.106 (0), *P* = 0.103 (1), *P* = 0.337 (2), *P* = 0.277 (≥3); Neutrophil, *P* = 0.414 (0), *P* = 0.028 (1), *P* = 0.043 (2), *P* = 0.030 (≥3); Lymphocyte, *P* = 0.065 (0), *P* = 0.925 (1), *P* = 0.431 (2), *P* = 0.629 (≥3); Monocyte, *P* = 0.288 (0), *P* = 0.162 (1), *P* = 0.106 (2), *P* = 0.083 (≥3); Eosinophil, *P* = 0.066 (0), *P* = 0.756 (1), *P* = 0.231 (2), *P* = 0.896 (≥3); HBV DNA, *P* = 0.512 (0), *P* = 0.875 (1), *P* = 0.635 (2), *P* = 0.003 (≥3); HBsAg, *P* = 0.777 (0), *P* = 0.868 (1), *P* <0.001 (2), *P* = 0.033 (≥3). Numbers in parentheses indicate coffee intakes (cups per day). Wilcoxon Signed Ranks Test was used to calculate the *P* values.
